# Metabolic Effects of Mulberry Leaves: Exploring Potential Benefits in Type 2 Diabetes and Hyperuricemia

**DOI:** 10.1155/2013/948627

**Published:** 2013-12-05

**Authors:** A. Hunyadi, E. Liktor-Busa, Á. Márki, A. Martins, N. Jedlinszki, T. J. Hsieh, M. Báthori, J. Hohmann, I. Zupkó

**Affiliations:** ^1^Institute of Pharmacognosy, Faculty of Pharmacy, University of Szeged, Eötvös u. 6, Szeged 6720, Hungary; ^2^Department of Pharmacodynamics and Biopharmacy, Faculty of Pharmacy, University of Szeged, Eötvös u. 6, Szeged 6720, Hungary; ^3^Department of Medical Microbiology and Immunobiology, Faculty of Medicine, University of Szeged, Dóm tér 13, Szeged 6720, Hungary; ^4^Unidade de Parasitologia e Microbiologia Médica, Instituto de Higiene e Medicina Tropical, Universidade Nova de Lisboa, Rua da Junqueira 100, 1349-008 Lisboa, Portugal; ^5^Department of Genome Medicine, College of Medicine, Kaohsiung Medical University, Shih Chuan 1st Rd. 100, Kaohsiung 807, Taiwan

## Abstract

The leaves of *Morus alba* L. have a long history in Traditional Chinese Medicine and also became valued by the ethnopharmacology of many other cultures. The worldwide known antidiabetic use of the drug has been suggested to arise from a complex combination effect of various constituents. Moreover, the drug is also a potential antihyperuricemic agent. Considering that type 2 diabetes and hyperuricemia are vice-versa in each other's important risk factors, the use of mulberry originated phytotherapeutics might provide an excellent option for the prevention and/or treatment of both conditions. Here we report a series of relevant *in vitro* and *in vivo* studies on the bioactivity of an extract of mulberry leaves and its fractions obtained by a stepwise gradient on silica gel. *In vivo* antihyperglycemic and antihyperuricemic activity, plasma antioxidant status, as well as *in vitro* glucose consumption by adipocytes in the presence or absence of insulin, xanthine oxidase inhibition, free radical scavenging activity, and inhibition of lipid peroxidation were tested. Known bioactive constituents of *M. alba* (chlorogenic acid, rutin, isoquercitrin, and loliolide) were identified and quantified from the HPLC-DAD fingerprint chromatograms. Iminosugar contents were investigated by MS/MS, 1-deoxynojirimycin was quantified, and amounts of 2-*O*-alpha-D-galactopyranosyl-1-deoxynojirimicin and fagomine were additionally estimated.

## 1. Introduction

Chronically elevated uric acid concentration in the serum has impact on human health at various levels; it frequently leads to the development of gout [1], and it is a relevant risk factor of several further chronic diseases. For example, it represents a significant risk for various cardiovascular and cerebrovascular diseases [2–4], and a tight connection between hyperuricemia and diabetes has recently been revealed; apparently, these two diseases mutually increase each other's incidence [5]. Hyperinsulinemia in type 2 diabetes can significantly increase reabsorption of uric acid in the proximal tubules, while its overproduction due to an increased activity of xanthine-oxidase usually also takes place [5, 6]. On the other hand, high uric acid levels might predict the development of metabolic syndrome consequentially leading to diabetes [7, 8] and can also increase severity of the already developed disease by leading to a higher incidence of certain diabetic complications [9]. The mechanism by which these metabolic states are interconnected seems yet to be clarified, although there is evidence that hyperuricemia, metabolic syndrome, and type II diabetes share the same causal origin in which insulin resistance would play a key role [5]. Anyhow, development of novel therapeutic agents targeting both diabetes and hyperuricemia appears to be a highly relevant strategy for overcoming difficulties attributed to the current therapeutic approaches. Appropriately chosen phytotherapeutics [10, 11], representing complex bioactivity profiles, might serve as excellent tools to fulfill this objective, either as monotherapy or in combination with already existing approaches.

Mulberry leaves are probably best known by their role in the silk production, but medicinal use of this drug also dates back at least two thousand years; it is already mentioned in the “Divine Farmer's Materia Medica” (pinyin: Shénnóng Běncǎo Jīng) written during the reign of the Han dynasty. In Traditional Chinese Medicine, leaves of *M. alba* possess sweet, slightly bitter, and slightly cold properties, and their primary uses are described as “to expel wind and heat from the lungs, as well as to clear the liver and the eyes” [12]. Anti-diabetic use of mulberry leaves had also been popular; moreover, this indication became part of the local traditional medicine wherever the tree has been naturalized [13–16]. Based on this, a large number of herbal preparations (including many food supplements) are worldwide available for diabetes treatment and easily accessible to everyone even via online shopping. This activity of mulberry leaves has been verified by a number of studies including several animal experiments [14–16] and a few human trials as well [17, 18], but, to our knowledge, the active constituents and their role in the activity still remain to be fully described. Nevertheless, a complex cocktail of various bioactive constituents is thought to be responsible for this activity [19], among which the role of iminosugars [18] and certain phenolics mainly chlorogenic acid and rutin [16] might be the most significant.

Furthermore, several traditional Chinese preparations utilize the branch of *Morus alba* for the treatment of gout, arthritis, and rheumatism [20]. Various constituents of the drug were found to have significant antihyperuricemic potential, including mulberroside A, a stilbene glycoside [21], and a number of flavonoids, primarily morin [22, 23].

Based on the above, our objectives were to explore the potential of *Morus alba* leaves as dual-target phytotherapeutics to prevent and treat both diabetes and hyperuricemia and to investigate whether a simple chromatographic fractionation can lead to the enrichment of the main bioactive constituents valuable for both therapeutic targets of interest.

## 2. Materials and Methods

### 2.1. Plant Material, Chemicals, and Reagents

The leaves of *Morus alba* were collected near Ásotthalom (nearby Szeged, Hungary) in May, 2007, and botanically identified by A. Hunyadi. A voucher specimen (MA052007) was deposited in the Institute of Pharmacognosy, University of Szeged, Szeged, Hungary. All chemicals, if otherwise not specified, were purchased from Sigma-Aldrich (Budapest, Hungary). Rutin (**2**) and isoquercitrin (**3**) were purchased from ChromaDex (Irvine, CA, USA) and Extrasynthése (Genay, France), respectively. Loliolide (**4**) was previously isolated from the dry leaves of *Morus alba* [19], and 1-Deoxynojirimicin (1-DN) was purchased from Wako Pure Chemical Industries (Osaka, Japan). HPLC grade methanol was obtained from Fischer Scientific; ultrapure water was obtained by using a Millipore Direct-Q UV3 equipment.

### 2.2. Extraction and Chromatographic Fractionation

2.5 kg of the dried and ground plant material was extracted by percolation with 30 L of 70% aqueous methanol and the solvent was evaporated under vacuum at 50°C to obtain 675.36 g dry extract (EX). 170 g of the dry material was further processed; it was dissolved in 1000 mL of water and extracted with 10 × 500 mL of n-butanol. After solvent evaporation, dry residue of the aqueous (FR-W) and organic phase (FR-B) was 78.4 and 88.09 g, respectively. The butanol phase was adsorbed onto triple amount (276 g) of silica (Kieselgel 60, Merck, Darmstadt, Germany) and administered on the top of a previously prepared silica column of 1840 g. A stepwise gradient of CH_2_Cl_2_, CH_2_Cl_2_ : EtOH (95 : 5, 9 : 1, 8 : 2, 7 : 3, 6 : 4, and 1 : 1), and EtOH was used, and one single fraction per solvent was collected. After the first fraction of 18 L, each following was of 10 L volume. After solvent evaporation, dry residues of the fractions were 13.72, 9.76, 4.48, 8.19, 10.52, 9.93, 5.50, and 10.87 g, respectively. Based on high similarities in their TLC fingerprints, the last three fractions were joined; hence, finally six fractions, referred to as FR1-6, were used for the experiments discussed here.

### 2.3. HPLC-DAD Analysis of Fractions FR1-6

Diode-array detected high performance liquid chromatography (HPLC-DAD) was performed on a gradient system of two Jasco PU2080 pumps connected to a Jasco MD-2010 Plus diode-array detector (DAD) and equipped with a Jasco AS-2055Plus autosampler (Jasco Co., Tokyo, Japan), by using a Zorbax Eclipse XDB-C8 (5 *μ*m, 4.6 × 150 mm) analytical column. Samples were dissolved in 30% of aqueous MeOH at 2 mg/mL, and 30 *μ*L of each was injected. Gradient elution was performed from 30 to 100% of aqueous MeOH in 13 minutes, kept at 100% for four more minutes, and returned to 30% at 17.1 min. Chromatograms were recorded for 24 min, and DAD data was collected from 200 to 650 nm. Baselines were corrected by subtracting the chromatogram of a single 30 *μ*L injection of 30% MeOH.

### 2.4. Quantitative Analysis of Compounds **1–4**

Single-point quantitative analysis was performed for the previously identified major constituents in their respective fractions such as chlorogenic acid (**1**), rutin (**2**), and isoquercitrin (**3**) in EX, FR-B and FR5-6 and loliolide (**4**) in FR2. Calibration curves were taken by analysing dilutions of a 1 mg/mL stock solution of the corresponding standard, at concentrations of 1, 0.5, 0.4, 0.3, 0.2, 0.1, and 0.05 mg/mL. *R* ^2^ values were 0.9970, 0.9990, 0.9904, and 0.9978 for the calibration lines of **1–4**, respectively. In case of FR5 and FR6, peaks of **2** and **3** were partially overlapping; deconvolution was performed by using Gaussian approximations with the Fityk 0.9.7 software (Free Software Foundation, Poland) and quantities were calculated based on the peak areas revealed this way.

### 2.5. Determination of Iminosugars

Iminosugar content was qualitatively investigated by thin-layer chromatography (TLC) in each fraction, by using DC-Alufolien Kieselgel 60F_254_ plates (Merck, Darmstadt, Germany) and CH_2_Cl_2_ : MeOH : NH_3_ (3 : 6 : 2, v/v/v) as solvent system. For quantitative analysis, an API 2000 MS/MS spectrometer was used with a Shimadzu autosampler and an electrospray ionization (ESI) interface set in positive mode. 1-DN, 2-*O*-alpha-D-galactopyranosyl-1-deoxynojirimicin (Gal-DN), and fagomine contents were investigated in FR-W, FR-B, and FR4-6. Injected samples were washed into the spectrometer with 50% aqueous MeOH at a flow rate of 200 *μ*L/min; temperature of the ion source was 300°C. Multiple reaction monitoring (MRM) was used with transitions of *m*/*z *164 → 69 for 1-DN, *m/z *326 → 164 for GAL-DN, and *m*/*z *148 → 86 for fagomine, according to literature data [24]. Parameter optimization and data acquisition and evaluation were performed by using the Analyst 1.5.1 software. Calibration line of 1-DN (*R* ^2^ = 1.0000) was obtained by means of six measurement points in triplicates.

### 2.6. *In Vitro* Assay on Xanthine Oxidase (EC 1.17.3.2) Inhibition

The activity of the enzyme was calculated from the increase of uric acid concentration determined by microplate-based kinetic photometry [25]. Briefly, the absorption of uric acid generated from xanthine (50 *μ*M at start-up) was followed at *λ* = 290 nm for 125 s (Spectrostar Nano, BMG Labtech, Ortenberg, Germany). The activity of xanthine oxidase was described as the slope of the absorbance versus time curve and allopurinol was used as positive control. Stock solutions prepared with dimethyl-sulfoxide were used for the *in vitro* assays and any substantial effect of the solvent was excluded. All *in vitro* assays were carried out in duplicates.

### 2.7. Determination of Free Radical Scavenging Activity by DPPH Assay *In Vitro*

The activity for scavenging 1,1-diphenyl-2-picrylhydrazyl (DPPH) radicals was tested for each fraction as described earlier [26]. Briefly, different amounts of the samples were added to 0.1 mM DPPH dissolved in ethanol. The mixture was shaken and allowed to stand for 30 min, and then the absorbance of the solution was measured at *λ* = 517 nm. trolox (6-hydroxy-2,5,7,8-tetramethylchroman-2-carboxylic acid), a water-soluble analog of vitamin E, was used as positive control.

### 2.8. Determination of Lipid Peroxidation Inhibitory Activity

The antioxidant properties of the fractions were additionally measured by means of inhibition of the autooxidation of unsaturated fatty acids present in animal brain tissue [27]. A lipid-rich fraction was prepared from the brains of male Sprague-Dawley rats (Charles River Laboratories, Budapest, Hungary; body mass: 250–300 g) by homogenization and centrifugation. The fatty acids in such a fraction get spontaneously oxidized during an incubation of 1 h at 37°C, and this oxidation can be inhibited by antioxidants. The oxidized products were determined by colorimetry at 532 nm after reaction with thiobarbituric acid. All *in vitro* experiments were carried out in duplicates and statistically evaluated. Sigmoid curves were fitted to the results of both antioxidant assays, and IC_50_ values were calculated by using GraphPad Prism 4 (GraphPad Software, San Diego, CA, USA).

### 2.9. Testing the Effect on the Glucose Consumption of Adipocytes

Effect of each fraction was tested on the *in vitro* glucose consumption of adipocytes as published before, at concentrations of 200 *μ*g/mL [19]. Briefly, 3T3-L1 preadipocytes (5 × 10^5^ cells, BCRC no. 60159; Bioresource Collection and Research Center, Taiwan) were seeded and cultured in 10% CS DMEM containing 5.5 mM D-glucose. The cells were induced to differentiate, and, at day 3, samples dissolved in DMSO were added to the cells at 200 *μ*g/mL either in presence or absence of 0.32 *μ*M insulin. 24 h changes in the glucose contents of the testing media were recorded and compared to those before the addition of the samples.

### 2.10. Animal Studies

Animals were treated in accordance with the European Communities Council Directives (86/609/ECC) and the Hungarian Act for the Protection of Animals in Research (XXVIII.tv.32.§). Experiments involving animal subjects were carried out with the approval of the Hungarian Ethical Committee for Animal Research (registration no. IV./01758-2/2008). Male Sprague-Dawley rats (Charles River Laboratories, Budapest, Hungary; body mass: 180–200 g; 8 animals in each group) were housed in temperature (20–23°C), humidity (40–60%), and light (12 h of light, 12 h of dark) regulated rooms, with tap water and rodent food (Bioplan, Isaszeg, Hungary) intake available *ad libitum*. The tested extracts were orally administered in 0.25% methylcellulose containing 2% Cremophor EL using a dosing volume of 5 mL/kg. Three doses of all extracts (30, 60, and 120 mg/kg) were selected for daily treatment for 3 consecutive days and each treatment was performed after 16 hours of fasting. One-way analysis of variance (ANOVA) with Dunnett's multiple comparison test by GraphPad Prism 4 was used for statistical evaluation of all *in vivo* experiments.

### 2.11. Determination of Antihyperuricemic, Antihyperglycemic, and Antioxidant Properties of the Fractions *In Vivo*

In order to minimize the number of animals, antihyperuricemic, antihyperglycemic, and antioxidant properties effects were determined from the same groups of rats. Experimental hyperuricemia model induced by uricase inhibitor potassium oxonate was utilized to assess the antihyperuricemic properties of the tested fractions [28]. 250 mg/kg potassium oxonate was suspended in 0.25% methylcellulose and administered intraperitoneally at the time of the third oral treatment. 50 mg/kg of allopurinol, a clinically used antigout drug, was orally administered as a positive control. One hour later rats were orally treated with 2.5 g/kg starch suspension in a dosing volume of 5 mL/kg in order to characterize the antihyperglycemic effects of the fractions [25]. After another one hour, venous blood samples were obtained from the tail, and the plasma glucose concentrations were determined by a commercially available kit (Reanal, Budapest, Hungary) based on the glucose oxidase-peroxidase method [29]. Immediately after blood sampling, rats were anesthetized in 4% isoflurane and additional blood samples were collected by cardiac puncture for determination of uric acid concentration and total antioxidant capacity. Since inhalational anesthetics are reported to substantially elevate the blood glucose level, separate blood samplings were needed for determination of all the planned parameters [30]. Serum samples were prepared by centrifugation and stored at –70°C until the analyses. All determinations by colorimetric uric acid (BioAassay Systems, Hayward, CA, USA) and antioxidant (Sigma-Aldrich, Budapest, Hungary) assay kits were performed in duplicates according to the manufacturers' suggestions. Separate groups orally treated with glibenclamide and trolox (10 mg/kg for both) were included as reference for the antihyperglycemic and antioxidant assays, respectively.

## 3. Results and Discussion

### 3.1. Chemical Composition of the Fractions Obtained

**Figure 1:**
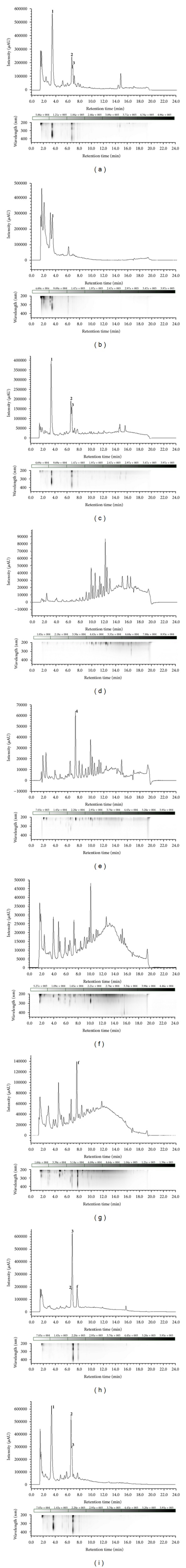
HPLC-DAD fingerprints of the samples and the corresponding maximum absorbance chromatograms from *λ* = 200 to 450 nm. (a) Crude extract (EX), (b) FR-W, (c) FR-B, and (d)–(i) FR1-FR6, respectively. Marked peaks represent chlorogenic acid (**1**), rutin (**2**), isoquercitrin (**3**), loliolide (**4**), and an unidentified flavone derivative (**f**).

The fractions, obtained by solvent-solvent extraction and a rough chromatographic separation of the organic phase on silica, represented fundamentally different chemical compositions (Figure 1). As expected, highly water soluble, hydrophilic compounds (including iminosugars of the plant, see below) remained in the water phase (Figure 1(b)), while most of the still polar chlorogenic acid (**1**) could already be detected in the organic phase (Figure 1(c)) along with vast majority of the supposedly “drug-like” secondary metabolites of mulberry leaves. These would be the compounds with the highest probability for therapeutic value; mainly the too low log *P* value typically results in poor penetration through membranes (iminosugars, a constituent group of possible exception to this, are discussed below). As seen from the DAD fingerprints, FR1 (Figure 1(d)) and FR2 (Figure 1(e)) mostly contained compounds with lower wavelengths of UV absorbance maxima probably including several terpenoids and/or phenylpropanes [19]. The dominant peak of FR2 was detected and quantified as loliolide (**4**) (0.33%), a monoterpene lactone we have recently reported from *M. alba* leaves [19]. Of all fractions, FR3 (Figure 1(f)) contained the smallest amount of UV absorbing material; its main constituents remained unidentified. Based on its UV spectrum and retention time, chief constituent of FR4 (Figure 1(g)) is suggested to be a flavone aglycone (3.42–3.50%, expressed in equivalents of isoquercitrin (**3**) or rutin (**2**)), while **3** (2.42%) was also present in this fraction. FR5 (Figure 1(h)) contained the majority of **3** (9.79%) along with a smaller amount of **2** (3.77%), while the previously mentioned unidentified flavonoid could still be detected. FR6 (Figure 1(i)), the most polar fraction obtained from the column chromatography, contained **1** (8.99%), **2** (10.41%), and **3** (5.64%) as major UV active constituents.

**Figure 2:**
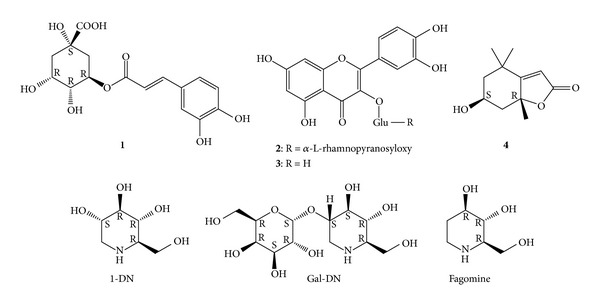
Chemical structures of the compounds identified in the fractions: chlorogenic acid (**1**), rutin (**2**), isoquercitrin (**3**), loliolide (**4**), 1-deoxynojirimycin (1-DN), 2-*O*-alpha-D-galactopyranosyl-1-deoxynojirimycin (Gal-DN), and fagomine. Glu: *β*-D-glucopyranosyloxy.

Both the extract and the fractions contained very small amounts of iminosugars. By means of MS/MS, 1-DN contents were found as low as 0.2694‰ (EX), 0.3007‰ (FR-W), 0.0742‰  (FR-B), ~0.016‰  (FR4; around detection limit), 0.0558‰  (FR5), and 0.2770‰ (FR6). On the other hand, slope of the calibration lines of GAL-DN and fagomine is around 13 and 6 times higher, respectively, as compared to that of 1-DN, based on a recent publication using the same MRM transitions with ESI-MS/MS [24]. This allowed only a rough estimation on the quantity of these compounds by using our calibration obtained for 1-DN. Amounts of GAL-DN were about 0.02‰ (EX), 0.03‰ (FR-W), 0.003‰ (FR-B) and 0.009‰ (FR6), and trace amounts of this compound were detected in FR4 and FR5. Fagomine contents could be estimated as around 0.03‰ (EX), 0.04‰ (FR-W), 0.01‰ (FR-B), 0.01‰ (FR5) and 0.03‰ (FR6), with a trace amount of this compound also present in FR4. Structures of the compounds identified from the fractions are shown in Figure 2.

### 3.2. Inhibition of Xanthin Oxidase *In Vitro*

All fractions were tested at the final concentration of 5 *μ*g/mL. The enzyme activity determined in the solvent-treated condition was considered 100% and all other conditions were compared to that control. None of the prepared fractions exerted any substantial action on the enzyme xanthine oxidase, while, as expected, the reference compound allopurinol resulted in a nearly complete (>90%) inhibition of the enzyme activity at 5 *μ*g/mL, and its IC_50_ value was determined as 1.03 *μ*g/mL. Based on these results, XO inhibition does not seem to be an important mechanism for neither the antihyperuricemic nor the antioxidant activity of mulberry preparations.

### 3.3. *In Vitro* Antioxidant Properties

Two different bioassays were utilized to investigate the *in vitro* antioxidant properties of the fractions: DPPH assay and lipid peroxidation (LOX) assay. Even though the results obtained by these two methods are frequently parallel, certain agents can exhibit substantial differences in these assays [31]. Generally, a compound effective in the DPPH assay can be considered as a free radical scavenger functioning in an organic solvent against a chemically pure molecule (i.e., the DPPH radical). LOX assay, on the other hand, is performed in a more complex *ex vivo* biological system containing lipids with unsaturated fatty acids; a substance active in this assay may protect these lipids from the spontaneous oxidation in aqueous conditions.

DPPH assay was performed with the concentration range of 0.001–0.15 mg/mL to characterize the free radical scavenging capacity of the prepared fractions. Fractions FR1 and FR2 exhibited no substantial activities in the utilized concentrations. Fractions FR3 and FR4 were moderately active, while FR5 and FR-W showed the highest potencies. FR6, containing significant amounts of phenolic compounds (compounds **1–3**), was equipotent with the reference agent trolox. In agreement with these, lipid peroxidation assay showed that the antioxidant capacities of fractions FR3 and FR6 were close to that of trolox, while FR5 and FR-W were slightly less active. Fractions FR2 and FR4 exhibited moderate activities, and, similarly to the case of the DPPH assay, FR1 was inactive. Results of both assays are summarized in Table 1.

### 3.4. Effect on the *In Vitro* Glucose Consumption of Adipocytes

Although some minor changes could be observed in this bioassay, no statistically significant activities were found. This was somewhat surprising, since a lipophilic fraction, obtained by a simple solvent-solvent distribution from the hot water extract of the same plant collection, was previously found to exert strong effect in this test [19]. The most likely explanation is the low relative amount of the constituents responsible for this effect, based on which we can also conclude that increasing the glucose consumption of adipose tissue has little importance in the complex anti-diabetic activity of less processed phytotherapeutics originated from mulberry leaves.

### 3.5. Determination of the Antihyperuricemic Effects of the Fractions *In Vivo*

**Figure 3:**
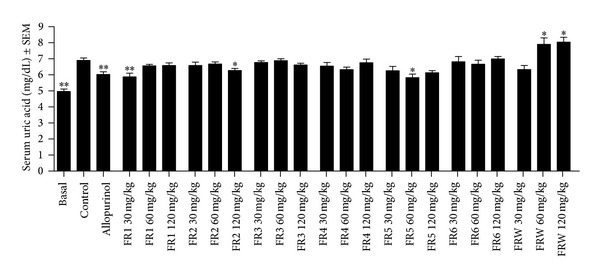
*In vivo* antihyperuricemic activity of the fractions obtained. Hyperuricemia was induced by a single administration of K-oxonate, 50 mg/kg of allopurinol was used as positive control, and samples were tested at 30, 60, and 120 mg/kg. ∗ and ∗∗: *P* < 0.05 and 0.01, respectively, as compared to the negative control by means of one-way ANOVA followed by Dunnett's multiple comparison test; *n* = 8.

The serum uric acid concentration in rats is considerably low because it is metabolized into allantoin by the enzyme uricase. Therefore, uric acid accumulation was induced by a single administration of K-oxonate for the *in vivo* investigation of the antihyperuricemic properties of the fractions. Results are shown in Figure 3. Allopurinol (50 mg/kg intraperitoneally), used as positive control, substantially and significantly decreased the accumulation of uric acid. The lowest dose (30 mg/kg) of FR1, the highest dose (120 mg/kg) of FR2, and 60 mg/kg of FR5 exerted antihyperuricemic actions comparable to that of allopurinol. Unexpectedly, treatment with FR-W (60 and 120 mg/kg) resulted in an elevation of serum uric acid levels. No clear dose-response relationships were detected for some of the fractions.

The renal excretion of uric acid is a complex and species-dependent procedure involving its glomerular filtration, tubular secretion, and tubular reabsorption. Since these processes can independently be modulated with exogenous substances, the overall action of a drug is frequently biphasic. Aspirin at dosages of >3 g/day promotes uricosuria by inhibition of the reabsorption while lower dosages (1-2 g/day) may cause uric acid retention, presumably by interfering with the tubular secretion [32]. This, together with the chemical complexity of the fractions, could provide a simple explanation for the unclear dose-response relationships observed in certain cases; several interactions between various constituents of the fractions might take place, in which the active components would influence these renal functions differently.

### 3.6. Determination of the Serum Antioxidant Capacity

**Figure 4:**
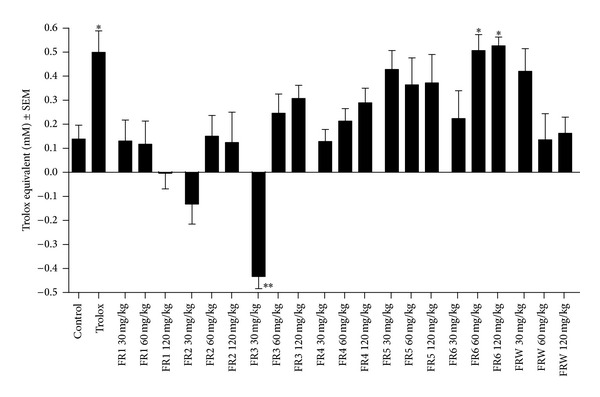
Effects on the serum antioxidant capacity *in vivo*. *P < 0.05 as compared to the negative control by one-way ANOVA followed by Dunnett's multiple comparison test; *n* = 8.

The antioxidant capacities of the serum samples of the treated animals were determined by means of a photometric assay in which 2,2′-azino-bis(3-ethylbenzthiazoline-6-sulfonic acid) (ABTS) is converted into a chromogen radical cation (ABTS^●+^). The generation of radical cation is suppressed by antioxidants and can be detected as a decrease of color intensity; results of this assay are shown in Figure 4. Treatment with trolox (10 mg/kg) resulted in a substantial increase in the antioxidant capacity (expressed as trolox equivalents) but most of the tested fractions failed to induce significant change in this serum parameter. The higher doses of FR6 (60 and 120 mg/kg) exhibited similar antioxidant effects to that of trolox, which effect can most likely be attributed to the high chlorogenic acid (**1**), rutin (**2**), and isoquercitrin (**3**) content of this fraction. On the other hand, 30 mg/kg of FR3 decreased the oxidative status of the serum, indicating a prooxidant potential for certain constituents.

### 3.7. Determination of the Postprandial Plasma Glucose Levels

**Figure 5:**
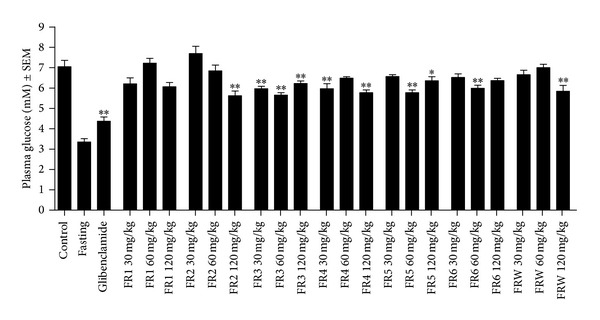
Effects on the postprandial hyperglycemia of normal rats after starch loading. ∗ and ∗∗: *P* < 0.05 and 0.01, respectively, as compared to the negative control by one-way ANOVA followed by Dunnett's multiple comparison test; *n* = 8. The fasting blood glucose level was determined in preliminary experiments, and its dataset was not included in the statistical evaluation.

Treatment with the positive control glibenclamide (10 mg/kg) resulted in a substantial and significant decrease of the postprandial plasma glucose levels obtained in the starch load model. FR1 was inactive up to 120 mg/kg, while FR3 exhibited antihyperglycemic action at all applied doses. Fractions FR2, FR-W, and FR5 were effective at 120, 60, and 120 mg/kg, respectively. Fractions FR4 and FR6 exhibited some antihyperglycemic properties but no clear dose-response relationships were observed; results are shown in Figure 5. It is worthy to note, however, that the present *in vivo* model investigated the effect of a single-dose administration on the postprandial hyperglycemia of normal rats after starch loading; therefore, these results can hardly be compared with those we obtained previously for a longer treatment in type 2 diabetic rats with *ad libitum* access to standard food [16].

## 4. Conclusions

The investigated extract of *M. alba* showed multiple beneficial bioactivities in view of both targeted chronic metabolic diseases. It can also be concluded, based on the different effects exerted by the fractions, that separate compound groups are responsible for the individual actions, which have a significant potential for a positive combined effect.

FR1, FR2, and FR5 are of interest in view of the antihyperuricemic activity as potential uricosuric agents. FR2–6 were all found valuable for their antihyperglycemic activities, but FR3 was the strongest exerting significant effects at all administered doses. Somewhat unsurprisingly, FR6, containing most of the phenolic constituents, was found to be the strongest antioxidant both *in vitro* and *in vivo*, which makes this fraction particularly useful against the high oxidative stress present in both diabetes and chronic hyperuricemia.

Furthermore, considering our original objectives, fractions of relatively low value could also be revealed. Although the water phase of the first solvent-solvent extraction (FR-W) could effectively scavenge DPPH radicals *in vitro* and showed at least a weak antihyperglycemic activity *in vivo *(possibly due to the low iminosugar content of our sample), this fraction was also found to significantly increase the plasma uric acid levels; hence, it is potentially unwanted in a well-designed mulberry preparation. FR1, the less polar fraction that was eluted with dichloromethane from the silica column, was found inactive in most bioactivity tests except for its non-dose-dependent antihyperuricemic activity at 30 mg/kg *in vivo*. This might be of interest for further research, but, due to the nearly 20% amount of this fraction by weight in the dry butanolic phase, removing this polarity range of constituents could as well be considered for increasing the overall therapeutic benefits of a phytotherapeutic product.

## Figures and Tables

**Table 1:** *In vitro* antioxidant activities as calculated from the DPPH assay and from the inhibition of the spontaneous lipid peroxidation.

Sample	DPPH	Lipid peroxidation
	EC_50_ (*μ*g/mL)	IC_50_ (*μ*g/mL)
FR-W	72.2	71.0
FR1	—^a^	—^a^
FR2	—^a^	138.0
FR3	186.0	47.3
FR4	245.0	114.0
FR5	55.0	77.7
FR6	21.7	55.8
Trolox	21.8	42.5

Trolox: positive control in both cases.

^a^Exhibited no substantial activity in the applied concentration range.
